# Review of Non-Invasive Imaging Technologies for Cutaneous Melanoma

**DOI:** 10.3390/bios15050297

**Published:** 2025-05-07

**Authors:** Luke Horton, Joseph W. Fakhoury, Rayyan Manwar, Ali Rajabi-Estarabadi, Dilara Turk, Sean O’Leary, Audrey Fotouhi, Steven Daveluy, Manu Jain, Keyvan Nouri, Darius Mehregan, Kamran Avanaki

**Affiliations:** 1 Department of Dermatology, University of California Irvine, Irvine, CA 92617, USA; luke.horton2@med.wayne.edu; 2 Department of Dermatology, Wayne State University School of Medicine, Wayne State University, Detroit, MI 48202, USA; jfakhour@med.wayne.edu (J.W.F.); dturk@med.wayne.edu (D.T.); soleary1@alumni.nd.edu (S.O.); sdaveluy@med.wayne.edu (S.D.); dmehregan@wayne.edu (D.M.); 3 The Richard and Loan Hill Department of Biomedical Engineering, University of Illinois at Chicago, Chicago, IL 60607, USA; rmanwar@uic.edu; 4 Department of Dermatology and Cutaneous Surgery, University of Miami Miller School of Medicine, Miami, FL 33136, USA; arajabi@med.miami.edu (A.R.-E.); knouri@med.miami.edu (K.N.); 5 Department of Medicine, University of Chicago, Chicago, IL 60637, USA; afotouhi@uchicago.edu; 6 Department of Dermatology, Memorial Sloan Kettering Cancer Center, New York, NY 10065, USA; jainm@mskcc.org; 7 Department of Dermatology, University of Illinois at Chicago College of Medicine, Chicago, IL 60607, USA

**Keywords:** skin, skin cancer, melanoma, melanoma imaging modalities

## Abstract

Imaging technologies are constantly being developed to improve not only melanoma diagnosis, but also staging, treatment planning, and disease progression. We start with a description of how melanoma is characterized using histology, and then continue by discussing nearly two dozen different technologies, including systems currently used in medical practice and those in development. For each technology, we describe its method of operation, how it is or would be projected to be most commonly used in diagnosing and managing melanoma, and for systems in current use, we identify at least one current manufacturer. We also provide a table including the biomarkers identified by and main limitations associated with each technology and conclude by offering suggestions on specific characteristics that might best enhance a technology’s potential for widespread clinical adoption.

## 1. Introduction

The worldwide incidence of cutaneous melanoma has increased over the last two decades [1,2,3,4,5,6]. Notably, the annual incidence has risen 4–6% in light skin populations, with a lifetime risk of one in thirty-three (3%) for Caucasians [7,8,9,10]. The increased detection of melanoma has likely played a role in the rise in diagnoses over the past 40 years [11,12]. While survival from the most common cancer types in the United States has increased from improvements in early detection and treatment, melanoma is still increasing in both morbidity and mortality each year [13,14]. Since 1975, the death rate has increased by 16%, with men being afflicted more than women [14]. Genetics and ultraviolet light exposure are known risk factors for melanoma [15], but the lack of availability of dermatologists in the county of residence is another independent risk factor [16,17]. Additionally, while the risk of melanoma diagnosis increases with age, with 65 being the average age of diagnosis, melanoma is still one of the most frequent cancer types diagnosed in individuals under the age of 30 [10,18]. Melanoma can appear anywhere on the body, but the most common locations are on the head and neck, back, and lower extremities [18].

Melanoma has the highest mortality of any skin cancer type, accounting for 75% of skin cancer deaths annually, despite making up only 1% of diagnosed skin cancers [10,19,20]. Lesion thickness is one the most important predictors of mortality of melanoma; therefore, early diagnosis is crucial for a better prognosis [21]. The current predicted five year survival rate for patients diagnosed with melanoma is greater than 99% for tumors excised before metastasis, decreasing to 75% with nodal metastasis, and to 49% for patients with distant metastasis [22].

The most widely accepted and utilized method of diagnosis relies on a skin exam adhering to ABCDE criteria, followed by biopsy and histopathologic analysis of suspicious lesions. This simple procedure relies heavily on dermatologist expertise; thus, its efficacy is variable [23]. Furthermore, since access to dermatologists varies widely by place of residence, patient demographics, and insurance status, the responsibility of performing full-body skin exams often falls upon primary care physicians who may or may not have recent training for melanoma detection [24]. These factors lead to both underdiagnosis and overdiagnosis. Underdiagnosis by primary care physicians causes melanomas detected by non-dermatologists to be thicker and later stage melanomas than those detected by dermatologists. Overdiagnosis leads to very high biopsy rates, with some centers reporting as many as 287 skin biopsies to diagnose one melanoma [25]. The increasing incidence of melanoma, the need to reduce costs of melanoma detection, as well as to form objective methods in skin cancer screening, has created a demand for an accurate, non-invasive, cost-effective, diagnostic imaging modality for melanoma. To that end, an explosion of detection techniques has been developed and adapted to the skin [26,27,28,29,30,31,32,33,34]. However, many challenges remain with regards to identifying and implementing prevention and early detection recommendations, understanding what drives the differences between dormancy and metastasis, and developing targeted therapy recommendations [35]. Appropriate imaging modalities may play a role in meeting these challenges. The connection between better screening and diagnostic information and prevention and early detection is straightforward. In addition, emerging imaging modalities may also play a part in identifying biomarkers of metastasis and assessing treatment efficacy sooner and non-invasively.

The histological subtype is important in the staging of melanoma, although it is not the only consideration. In melanoma, relevant prognostic biomarkers for staging include the tumor thickness (Breslow depth), Clark level, dermal mitotic rate per square millimeter, presence of lymphocytic invasion, degree of atypia, ulceration, tumor regression, presence of perineural or angiolymphatic invasion (tumor invasion of the dermis microvasculature), neurotropism, microsatellitosis, and margin assessment [36,37,38]. Tumor thickness and ulceration are important prognostic factors, with thickness being the most accurate tumor characteristic predictive of survival [39,40,41]. The tumor stage (T) is determined by the tumor thickness and presence of ulceration [38]. The nodal stage (N) is determined by the number of lymph nodes involved. This can be determined via sentinel lymph node biopsy (SNLB) and physical exam [42]. Although not included in the staging of melanoma, a high mitotic rate is associated with an increased risk of SLN metastasis [41,43,44]. The metastatic stage (M) of the disease is determined by the presence or absence of metastasis and the site of metastasis (skin, lymph nodes, viscera, lungs, etc.). Melanoma without metastasis is defined as either stage I or II, depending on the extent of vertical invasion. Either microscopic or gross metastasis to lymph nodes defines stage III melanoma. Stage IV melanoma is characterized by distant metastasis and elevated levels of serum lactate dehydrogenase (LDH) [38,45,46]. Stage 0 melanoma (in situ) is defined by the tumor cells being microscopically identified but confined to the epidermis, with no evidence that cancer has spread to the lymph nodes or distant sites [47].

Beyond lower morbidity and mortality for the patient, targeted early detection screening programs can be cost effective [48,49]. The advent of adjuvant treatments for advanced melanoma (e.g., one year of adjuvant pembrolizumab is estimated to cost over USD 160,000) emphasizes the potential cost savings to the healthcare system, should a comprehensive early detection screening program be implemented. Imaging modalities could also lead to non-invasive monitoring of the course of melanoma progression from diagnostics to differentiation of metastatic dormancy and progression, and even monitor response to therapy. In this review, we thoroughly describe the operation and strengths and shortcomings of over 20 imaging and spectroscopic modalities for melanoma screening, staging, treatment planning, and disease tracking by analyzing each modality for accuracy, reproducibility, cost, and current technology readiness level. This is not intended to serve as a scoping review of every clinical trial that has been performed using each of the described technologies but rather as an entry for familiarization with the breadth of technologies available for this challenging disease. For technologies still in development, we also assess their potential for widespread adoption.

## 2. Melanoma Characterization (Without Imaging)

### 2.1. Melanoma Formation and Subtypes

Melanoma begins in melanocytes, which are primarily found in the basal layer of the epidermis. Malignant melanocytes, that is, melanocytes with uncontrolled growth, form from genetic mutations. Melanomas typically follow a radial growth phase in the epidermis, followed by a vertical growth phase which leads to invasive spread, with different forms of melanoma progressing at different rates, many never going beyond radial growth, and some rapidly progressing to vertical growth [50].

Melanoma has a wide variety of presentations, and histopathological features vary by type of melanoma [51,52]. Histopathological features of superficial spreading melanoma (SSM) include asymmetry, poor circumscription, and lack of cellular maturation. In SSM, malignant melanocytes, large, atypical epitheliods with large nuclei, can spread as single cells or nests. The presence of melanocytes above the basal layer is known as Pagetoid spread; this is common in SSM but is also seen, to a lesser extent, in benign nevi. Melanocytic nests in SSM will display dyscohesion; they will look like they are falling apart. Nodular melanoma (NM) often displays a thinning of the epidermis and dermis, and histopathological features can include a nodular confluence of atypical melanocytes that are epitheloid or spindled with frequent and often atypical mitoses. Balloon cells are also seen. In the vertical growth phase, the dermal component of NM looks very similar to the dermal component of SSM. By contrast, lentigo maligna melanoma (LMM) is a slow-growing form of melanoma that can be identified by the proliferation of atypical melanocytes in the epidermal basal layer, where the atypical melanocytes are polygonal with atypical nuclei and the epidermis is often described as atrophic. In the dermis, melanocytes are hyperchromatic, typically small, and may be spindle-shaped or multinucleated. As LMM progresses, nodules are formed in the dermis and the atypical melanocytes proliferate along the dermal epidermal junction (DEJ) and down cutaneous appendages. Unlike in SSM and NM, in LMM, Pagetoid spread is not common, but the dermis shows solar elastosis (yellowing of skin due to sun damage). Acral lentiginous melanoma (ALM) is usually found within nail beds. Melanocytes present as nests and single cells along the DEJ. Pagetoid upward migration is widespread and melanocytes in the epidermis resemble those seen in LMM. Dermal invasion often tracks down eccrine structures and aggregates around blood vessels. Large DEJ nests of atypical melanocytes can be found.

### 2.2. Pathological and Histopathological Analysis of Melanoma

The analysis of a biopsied skin sample starts with fixing the tissue in formalin, embedding it in paraffin, and thinly slicing it. The slices are mounted on glass slides and stained, usually by hematoxylin and eosin (H&E). This staining enables pathologists to analyze tissue at the cellular level to identify the presence, type, and stage of melanoma.

All of features identified above can be seen by H&E staining, but melanoma is heterogeneous and there are histological mimics of melanoma [53]. In fact, differences in the interpretation of morphological features can lead to high levels of interobserver variation: in one study, 17% of diagnoses were recommended to be reclassified when reviewed by a specialist panel (both false positives and false negatives identified) [54]. This has led to incorporation of immunohistochemical (IHC) stains of melanocytic markers and proliferative markers. Melanocytic markers include S-100, Melan-A, and HMB-45 [55]. S-100 is a calcium-binding protein expressed by melanoma cells; Melan-A is a melanoma-associated antigen, and HMB-45 is a monoclonal antibody that targets the premelanosome protein gp100 [56]. The most commonly used proliferation marker is Ki-67, which is highly elevated in the most aggressive melanomas [57]. The combination of morphological features and molecular features increases the melanoma detection accuracy.

## 3. Review Method

**Figure 1:**
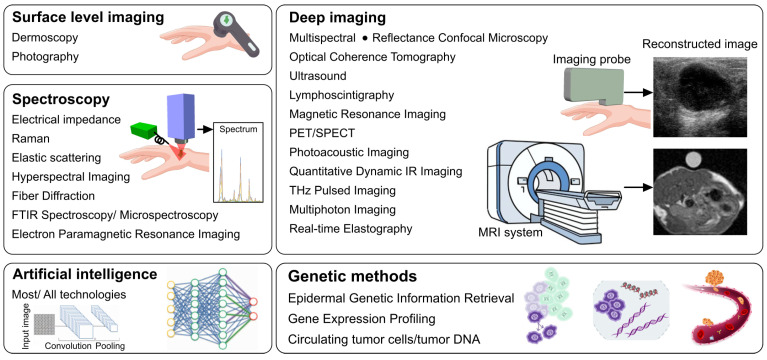
Overview of technologies included in this review.

To investigate the emerging non-invasive techniques in melanoma diagnosis, staging, and monitoring, we utilized PubMed and Google Scholar and searched “melanoma” with each of the following more than 20 modalities (Figure 1): digital photography (including total body photography), dermoscopy, hyperspectral imaging, multispectral imaging, electrical impedance spectroscopy, electron paramagnetic resonance spectroscopy, reflectance confocal microscopy, photoacoustic imaging (and optoacoustic imaging), optical coherence tomography, non-interferometric photoacoustic remote sensing microscopy, Raman spectroscopy, elastic scattering spectroscopy, real-time elastography, terahertz pulsed imaging, multiphoton imaging, ultrasound (including high-frequency ultrasound), magnetic resonance imaging, positron emission tomography, single photon emission computed tomography, fiber diffraction, and Fourier transform infrared spectroscopy (and microspectroscopy), restricting our search to studies published between 1995 and 2024. It should be noted that as we delved into the subject, we realized the value in including sensing technologies and spectroscopy methods not commonly considered “imaging”. These were incorporated into the review in order to cover the full range of non-invasive techniques involved in melanoma diagnosis, staging, and monitoring.

**Figure 2:**
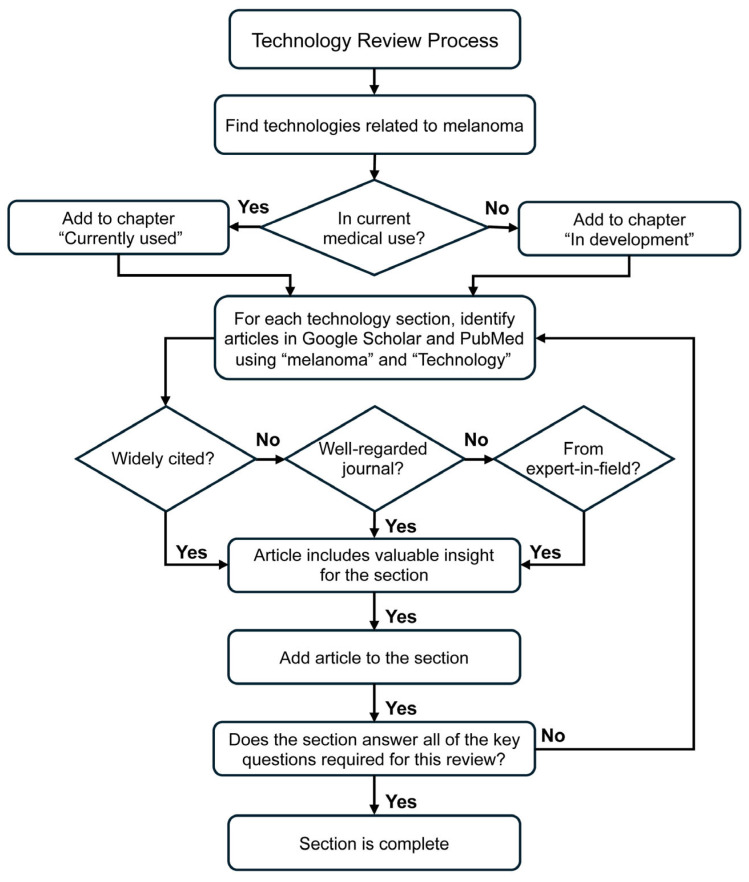
Flowchart describing process for inclusion of articles in this review.

Our searches (Figure 2) generated over 400,000 articles. For each search, we identified a selection of articles from high-impact journals and from leaders in the field, with the highest citation counts and/or that otherwise best exemplified the use of the technology for melanoma staging, screening, and monitoring, in our opinion. We discuss the principles of each modality, followed by a summary of their strengths and limitations. The first section describes imaging modalities commonly used in daily medical practice and those with more robust data in melanoma diagnosis and management; the second section describes technologies still under development.

## 4. Imaging Modalities Currently Used in Medical Practice

### 4.1. Photography

**Figure 3:**
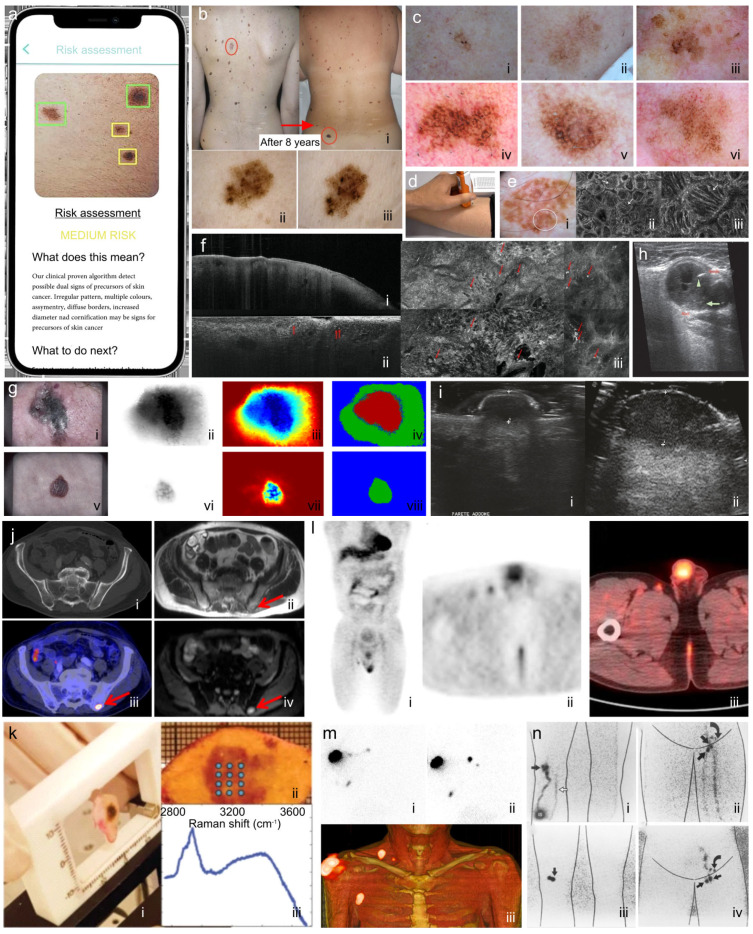
Images from technologies currently used in medical practice. (**a**) Smart phone image analysis, courtesy of [59]. (**b**) Total body digital photography (**i**–**iii**), courtesy of [58]. (**c**) Dermoscopy of lentigo maligna (**i**–**vi**), courtesy of [74]. (**d**) Electrical impedance spectroscopy protocol. Image courtesy of [243]. (**e**) Superficial spreading melanoma in situ shown by (**i**) dermoscopic image, (**ii**) RCM mosaic image, and (**iii**) RCM individual image. White arrows show biomarkers of melanoma, courtesy of [109]. (**f**) OCT images of melanoma: (**i**) vertical image by conventional OCT, (**ii**) verticle image of melanoma by LC-OCT, and (**iii**) horizontal image showing irregular honeycomb pattern with atypical melanocytic cells, courtesy of [144]. (**g**) Hyperspectral imaging of a melanoma (**i-iv**) and a benign nevus (**v**–**viii**). Left to right: photographic image for lesion localization in RGB, raw mosaic image, image after preprocessing, and classification result (red, malignant melanoma), courtesy of [160]. (**h**) Fine needle biopsy under ultrasound guidance, courtesy of [244]. (**i**) Measurement of primary cutaneous melanoma by HFUS (**i**,**ii**), courtesy of [174]. (**j**) Comparison of (**i**) CT (lesion is not evident), (**ii**) MRI (rapid acquisition sequency) -lesion is evident, (**iii**) PET imaging of metastatic bone lesion is evident (**iv**) MRI (diffuse weighted imaging sequence) -lesion is evident, courtesy of [193]. (**k**) Raman spectroscopy (**i**,**ii**) of biopsied tissue generates (**iii**) Raman shift spectra. Image courtesy of [209]. (**l**) Malignant lymph node detected by PET/CT. Coronal (**i**), transaxial (**ii**), and PET/CT fusion (**iii**) images of foot after ingesting tracer. Image courtesy of [245]. (**m**) SPECT/CT image showing injection site (shoulder) and two sentinel lymph nodes. Static images acquired after 10 min (**i**) and 2 h (**ii**) post injection and (**iii**)volume-rendered SPECT-CT, courtesy of [223]. (**n**) Radiography images of tracer dye movement to identify sentinel lymph nodes imaged shown from calf to popliteal (**i**) and groin (**ii**) 10 min and one hour (**iii**,**iv**) after injection of tracer dye, courtesy of [226]. Key biomarkers associated with each modality are described in Table 1.

Methods of photography, including total body digital photography (TBDP) [58], mole mapping, various forms of 3D photography, and smartphone photography [59], are being increasingly utilized to diagnose melanoma [60,61,62,63,64]. Photography allows clinicians to study lesions visually over time and is a useful method to help diagnose melanoma in high-risk patients, including those with multiple dysplastic nevi, a family history of melanoma, or both [63]. Recent studies have described the utility of photography in detecting early melanomas [65,66], which have greater accuracy when combined with dermoscopy [67,68]. Digital photography only allows for the naked eye evaluation of lesions [69]. Moreover, photography can play an essential role in telemedicine, especially in rural settings [70]. A shortcoming of smartphone photography is that the cameras produce inaccurate colors when operating in automatic mode and use a filter which intensifies edges. These deficiencies can be eliminated through adjusting camera parameters manually or during post-production [64]. An important limitation of photography is that it only images the surface features of the lesions [65]. Photography may be more useful in older patients, as one study showed that less than 1% of new lesions were histologically confirmed to be melanoma in patients younger than 50 years old, while 30% of new lesions were melanoma for those older than 50 [71]. A recent review of TBDP studies found the method enhances the surveillance and detection of new lesions, and is less time-consuming, but did not outperform dermatologists and should be integrated with dermoscopy and dermatologist expertise [72]. Examples of image assessments by smartphone (Figure 3a) and by TBDP (Figure 3b) are included below. TBDP systems such as 3D Vectra are offered by Canfield Scientific (Parsippany, NJ, USA) and FotoFinder Systems GmbH (Bad Birnbach, Germany), among others.

### 4.2. Dermoscopy

Dermoscopy is performed with a relatively inexpensive device, the dermatoscope. This simple operator-dependent modality is well suited for the diagnosis of pigmented lesions [73,74]. Dermoscopy relies on color and structure to differentiate between melanoma and benign nevi [75]. Dermoscopy offers magnified (6×–100×) images of the lesion in the horizontal plane [76,77]. There are two main dermoscopy systems: (i) immersion, non-polarized contact dermoscopy, which uses fluid to improve contact between the lens and the lesion and decrease the light reflected by the stratum corneum, and (ii) polarized light dermoscopy, which uses a filter to block reflected light. Dual-mode dermoscopy incorporates both systems, allowing the physician to examine the superficial components of the skin in immersion mode and the deeper structures in the polarized mode [78]. A study by Benvenuto-Andrade et al. found that under polarized light, melanin looked sharper and darker and vessels were better visualized, making it more useful in identifying malignancies compared to non-polarized light [79].

With dermoscopy, the physician can apply a variety of algorithms to determine if a suspicious lesion should undergo biopsy, including the three-point method, seven-point checklist, Menzies method, pattern analysis, and others [69,80,81]. General features of melanoma on dermoscopy include asymmetry, numerous colors, negative pigment network, chrysalis structures, blue–white veil, and regression structures, among others [77,82].

Numerous studies have described a significant improvement in the diagnostic accuracy of melanoma through the use of dermoscopy [69,83,84,85,86]. Sensitivities of 85–95% and specificities of 73–86% for the dermoscopic identification of melanoma were reported in a recent scoping review article [80]. A retrospective study showed that the excision ratio of benign–malignant lesions decreased from 18:1 in the pre-dermoscopy era to 4:1 with the use of dermoscopy in a specialist setting [87]. Super-high-resolution dermoscopy has recently been implemented with 400× magnification (typical dermoscopy utilizes 20× magnification). Super-high-resolution dermoscopy was able to identify individual pigmented cells in malignant melanoma and other features that could help differentiate melanomas from benign nevi [88]. A limitation of this modality (super-high-resolution dermoscopy) is the small field of view and the potential for inter-operator variability in analysis.

Dermoscopy is a quick, non-invasive method to increase diagnostic accuracy in real-time for clinicians, but it relies on biopsy and histologic analysis to confirm a diagnosis of melanoma [89,90]. Further, dermoscopy remains limited as a diagnostic tool, as certain melanoma subtypes, such as desmoplastic melanoma, often present devoid of characteristic dermoscopic features. Moreover, because dermoscopic features vary widely on different body locations, its efficacy depends heavily on the skill of the practitioner [89]. To resolve this problem, computer-aided digital dermoscopic image analysis is being refined to improve diagnostic performance for all practitioners [81,90,91,92,93]. Sequential digital dermoscopic imaging (SDDI) is a technique used to store and retrieve dermoscopic images for comparative analysis over time. SDDI monitors lesion evolution, but it requires frequent visits, often performed every three months. Encouragingly, SDDI has been shown to detect melanoma earlier in its progression [94,95,96,97]. Dermoscopic images of lentigo maligna are shown in Figure 3c. Manufacturers of dermoscopic equipment include DermLite (Aliso Viejo, CA, USA) and Canfield Scientific, (Parsippany, NJ, USA) among others.

### 4.3. Electrical Impedance Spectroscopy

Skin lesions can be analyzed by measuring the electrical impedance of cutaneous structures and comparing the value to nearby healthy skin, since pathogenic alterations in tissue influence the ability of cells to conduct electricity [98]. Electrical impedance spectroscopy (EIS) applies an alternating electric current to the skin and can detect changes in cell size, orientation, shape, and structure of the cell membrane. EIS does not create images of the skin; rather, it uses algorithms to provide a score based on the resistance [99]. Electrical resistance is measured over a range of four different depths (different colors) and ten permutations at different frequencies (1.0 kHz to 2.5 MHz), utilizing a safe voltage (i.e., 150 mV and 75 micro-A) [100]. The device is intended for lesions measuring between 2 mm and 20 mm in diameter [98].

EIS is a simple, fast, and safe procedure used to increase diagnostic accuracy. Figure 3d shows a typical implementation of EIS on a suspected lesion. EIS can assess lesions deemed suspicious on clinical and dermoscopic examination, with high reported sensitivity in detecting melanoma [101]. Various classification algorithms of EIS imaging have been developed [100,102]. A recent review article found reported sensitivities of 100–95% and specificities of 69.5% to 58.6% [103]. While EIS may be useful in detecting early melanomas and monitoring lesions over time, it has a high false-positive rate, and inflammation, ulceration, or scar tissue may further limit its validity [99,101,104]. EIS should be performed by physicians who are trained to clinically detect skin cancer because benign lesions, such as seborrheic keratoses, are frequently inaccurately classified as malignant by EIS [99,101]. One of the most widely known EIS devices is the Nevisense system (SciBase AB, Stockholm, Sweden). The manufacturer noted that an update fee schedule for the use of Nevisense for melanoma detection was published in 2023 [105].

### 4.4. Reflectance Confocal Microscopy

Reflectance confocal microscopy (RCM) provides cellular-level high-resolution images of the epidermis and papillary dermis in real-time [106,107,108]. Superficial spreading melanoma in situ (dermoscopic and RCM images) is shown in Figure 3e [109]. RCM has shown diagnostic potential in differentiating benign melanocytic lesions (nevi) from melanoma. RCM operates by directing a focused laser beam at the skin and capturing backscattered light from different tissue depths, using a spatial pinhole to eliminate out-of-focus signals, thus producing high-resolution, enface images of cellular structures in the epidermis and dermis with micrometer-scale optical sectioning. [89]. RCM is capable of presenting two-dimensional (*en face*) horizontal images of the skin up to a depth of a few hundred to several hundred μm, depending on the system configuration, including the light source wavelength. It is typically used to image from the top-most layer of the stratum corneum into the superficial (papillary) dermis [76,106,110,111,112]. These sequential depth images can be stacked into a three-dimensional rendering of the imaged area [106].

RCM relies on the intrinsic reflective structures found in the skin, such as free cytoplasmic melanin, melanosomes, and keratin, which provide a sharp contrast for near infrared (NIR) light sources [108,110]. RCM allows for near histological cellular resolution (0.5–1 µm lateral and 3–5 µm axial), providing the shape, distribution, and morphology of cells, as well as the visualization of the dermal–epidermal junction and vessels [107,113]. Additionally, this technique shows a good correlation with dermoscopic and histologic findings of malignancies such as basal cell carcinoma (BCC), squamous cell carcinoma (SCC), and melanoma, especially the LMM subtype [114]. It is particularly well suited for the examination of flat skin lesions and is used to define tumor margins and monitor responses to therapies [112,115].

Because RCM has higher specificity than dermoscopy, it leads to fewer unnecessary biopsies, which is particularly useful for the in vivo imaging of cosmetically sensitive areas such as the face and genital region [116]. Additionally, because the biomarkers for melanoma that RCM relies on are not pigment-based (e.g., atypical keratinocytes, pagetoid cells, changes in skin architecture), RCM has been used to detect both pigmented and amelanotic melanoma that are challenging to diagnose with dermoscopy [110,111,112]. Other uses of RCM include assessing margins of slow-growing LMM [117] and as an adjunct modality for lesions requiring re-excision [118]. Because RCM optical sectioning provides cellular-level image interpretation if widely used, it could significantly reduce the number of biopsies performed for in situ melanoma. In addition, RCM can be also used ex vivo on freshly excised tissue with slight laboratory processing (gentle flattening, moistening, with no need for tissue fixation or staining), and can be used in Mohs micrographic surgery to accelerate the definition of surgical margins [116].

A recent meta-analysis of 32 studies shows a pooled sensitivity and specificity of 92% and 70% for RCM [119]. Although RCM provides excellent resolution, significant dermatological expertise is needed to interpret RCM images for melanoma detection. For example, it is difficult to distinguish dendritic melanoma cells (pagetoid cells) from dendritic benign Langerhans cells, which can be seen in pigmented actinic keratosis and traumatized nevi, often leading to the overdiagnosis of melanoma [116,120]. Consequently, the diagnostic accuracy of RCM depends upon the skill of the user, where expertise is acquired through extensive training [98]. A major factor in the implementation of RCM is that individual RCM systems can cost >USD 100 k. This makes them too costly for most independent dermatology practices. Caliber ID (Rochester, NY, USA) is a manufacturer of RCM devices. RCM obtained billing codes in 2018 and is being integrated in clinics across the US.

### 4.5. Optical Coherence Tomography

Different configurations of optical coherence tomography (OCT) have been used in dermatology. These include swept source (SS) OCT, spectral domain (SD) OCT, dynamic (D) OCT, line-field confocal (LC) OCT, full-field (FF) OCT, high-definition (HD) OCT, and optical coherence microscopy (OCM) [121]. Depending on the configuration, OCT generates 2D and 3D images from backscattered light from within the tissue [122,123,124,125,126,127,128]. OCT imaging is based on endogenous scatterers and can image more significant volumes of skin than RCM [18,73]. OCT has been used to image the stratum corneum of glabrous skin, the epidermis, papillary dermis, dermo–epidermal junction, blood vessels, sweat glands, and hair follicles [89,122,129]. SS-OCT has a deeper penetration depth but a lower resolution than OCM, HD-OCT, or LC-OCT. A typical implementation of SS-OCT images uses a field of view of 6.0 × 6.0 mm^2^, an axial resolution of 5–10 µm, a lateral resolution of 7.5–15 µm, and a penetration depth of up to 2 mm [18,124,130,131,132]. This places SS-OCT resolution between RCM and high-frequency ultrasound. SS-OCT provides architectural details within tissue with near-cellular clarity, superior to high-frequency ultrasound and with better penetration depth than RCM, yet SS-OCT resolution is not sufficient to distinguish individual cells, limiting its usefulness for distinguishing between pigmented benign and malignant lesions such as dysplastic nevi and melanoma [124,133]. SS-OCT cannot characterize melanoma from visually identifiable features [133]. The low penetration depth of under 2 mm prevents OCT from imaging the breadth of more advanced tumors. Further, SS-OCT cannot delineate cellular features and relies on distinct architectural pattern recognition; its usefulness is therefore limited in the diagnosis of cutaneous melanoma [89,134]. To overcome this limitation, Turani et al. proposed a computational method for the analysis of OCT images. One study reported a high sensitivity (97%) and specificity (98%) in differentiating melanoma from benign nevi [133]. Another research group utilized deep learning to train a computational kernel to differentiate melanoma and benign nevi in mice, achieving 98/99% specificity and sensitivity with their model [135].

D-OCT is based on speckle variance and can visualize skin microvasculature and detect blood vessels within specific lesions by detecting motion and blood flow—these images may enhance the diagnostic accuracy of melanoma [136]. HD-OCT has a resolution of 3 μm for both lateral and axial imaging, allowing for the visualization of individual cells [137]. The penetration depth is 0.5 to 1.0 mm and the field of view is 1.8 × 1.5 mm^2^. For example, HD-OCT has been used to measure melanoma tumor thickness for shallow tumors relatively accurately, within an average error of 0.08 mm compared with histologic measurement [138]. A 2014 study of 64 patients, evaluating 93 melanocytic lesions (27 melanomas), indicated a sensitivity of 74.1% and specificity of 92.4% for HD-OCT [137]. Gambichler et al. indicated the accuracy of a melanoma diagnosis with HD-OCT depends upon tumor thickness and the existence of other suspicious lesions, as thin melanomas had a high false-negative rate and dysplastic nevi had a high false-positive rate [137].

LC-OCT utilizes a supercontinuum fiber laser as a broadband spatially coherent light source, typically with a central wavelength of ~800 nm [139]. This configuration enhances resolution to ~1.5 μm but reduces penetration depth. However, it has been implemented with a three-dimensional cube that provides fully cellular resolution [139,140,141,142,143]. LC-OCT can visualize melanoma directly, including providing horizontal images similar to RCM in clarity [144]. OCM is an extension of OCT that integrates microscopy-level spatial resolution by combining OCT with high-numerical-aperture (NA) objectives [145,146]. OCM achieves high lateral resolution (1–2 μm) due to the use of microscope objectives: resolution can approach the cellular level. The penetration depth is 400–700 μm and the field of view is around 1 to 2 mm. OCM fused with pump–probe spectroscopy has demonstrated the ability to detect melanoma in a human skin sample [147]. Compared to SS-OCT, HD-OCT, LC-OCT, and OCM can differentiate between malignant and benign melanocytic lesions (nevi). One study of LC-OCT reported 93% sensitivity and 100% specificity [146]; studies are underway to validate their utility [148,149].

Each version of OCT has difficulty visualizing deeper structures as the lesion thickness increases, limiting the ability to image deep tumor invasion [149]. Further, OCT scanning devices are expensive, and the technology is considered experimental by most insurers and therefore not reimbursed. With the similarities in cost and design between RCM and OCT devices, there is hope that OCT will soon be reimbursed. Other configurations of OCT can be learned from more established disciplines that currently use OCT, including ophthalmology [121,150,151,152,153,154]. Conventional and LC-OCT images are shown in Figure 3f. There are several commercial OCT systems available, such as the SS-OCT system VivoSight (Michelson Diagnostics, Maidstone, UK), which is equipped with D-OCT capability, an HD-OCT system SkinTell (Agfa, Mortsel, Belgium), and the LC-OCT system DeepLive (Damae Medical Paris, France). Aquyre Biosciences has an FF-OCT system (CelTivity System) for histological applications.

### 4.6. Multispectral Imaging

Multispectral imaging (MSI) creates images of the epidermis and papillary dermis by using multispectral illumination to illuminate sub-surface pigmentation in lesions up to 2 mm thick [155,156]. MSI uses multiple wavelengths of visible and near-infrared light to illuminate a lesion. Chromophores in hemoglobin, melanin, and collagen absorb and the transmit energy that can be measured/imaged [157]. These images have been used to analyze melanoma, pigmented skin lesions, basal cell carcinoma, and skin color [156,158,159,160]. In 2012, Bekina et al. used MSI to analyze lesions at four different wavelengths: 450 nm to evaluate superficial layers, 545 nm to evaluate blood distribution, 660 nm to detect melanin, and 940 nm for deeper skin structures [161]. Figure 3g shows a comparative overview of the multispectral image analysis of a melanoma and a benign nevus [160].

In a 2011 multicenter, blinded study, Monheit et al. [162] investigated the effectiveness of an MSI system on 127 melanoma lesions (in vivo) and compared the results to an independent biopsy reader study performed by 39 dermatologists. They demonstrated a 98.4% sensitivity in comparison to the 78% sensitivity of the dermatologists. Additionally, on pigmented lesions biopsied to rule out melanoma, MSI demonstrated a 9.9% specificity in contrast to the 3.7% specificity by dermatologists. The study concluded MSI to be a safe and effective aid in the diagnosis of melanomas [162]. Similarly, a 2017 study by Fink et al. [163] analyzed the performance of MSI in the clinical setting. In the study, 360 pigmented skin lesions were observed by dermatologists using an MSI system called Melafind, which produces a score based on the probability of melanoma. Lesions with scores > 2 were considered suspicious of malignancy, but the decision to biopsy was made by the dermatologist. Of the 113 lesions biopsied, the sensitivity and specificity of MelaFind were 100% and 5.5%, respectively (68.5% specificity for the entire set of 360 lesions) [163]. Overall, both studies demonstrate the high sensitivity of MSI but low specificity. Although this imaging method is a useful tool to decide whether to biopsy or not, MSI does not have depth sectioning capability and as such depth information is limited; further, is it not designed to evaluate colorless amelanotic melanomas [155,157]. MSI is not indicated for use in the eyes, mucosal, subungual, palmar, or plantar (acral) anatomical areas [61].

MSI has also been extensively evaluated as a tool for improving pathology screening [164]. While it has demonstrated efficacy, frameworks for skin tissue classification and segmentation are not standardized and the method has not been widely adopted clinically. MelaFind (Strata Skin Sciences, Horsham, PA, USA) was one of the manufacturers of MSI which has discontinued the development and sales of its MSI product line, effective on 30 September 2017 [165].

### 4.7. Ultrasound and High-Frequency Ultrasound

Ultrasound transmits ultrasound waves into the tissue and uses the reflected sound wave to reconstruct an image of the internal structures. Conventional ultrasound (3.5–14 MHz) is used for measuring lymph nodes and classifying them as benign, suspicious, or malignant prior to fine needle aspiration (FNAC) biopsy [166]. Ultrasound analysis often relies on the Berlin morphological criteria to predict metastasis to sentinel nodes. The criteria include the presence of peripheral perfusion, loss of central echoes, and balloon-shaped lymph nodes [166]. Conventional ultrasound has greater depth penetration but less spatial resolution than ultrahigh and high-frequency ultrasound (HFUS) and is therefore not typically used for analyzing primary melanomas [167]. HFUS provides real-time, non-invasive, non-ionizing images of cross-section slices through the skin, in a similar orientation to histology or OCT [106,168]. Transducers of 20, 75, or 100 MHz have been developed, offering a resolution of 10–200 µm and a penetration depth of 1.5 to 10 mm, with higher frequencies having lower penetration depths, but higher resolution, up to histological resolution [169,170]. HFUS has been utilized to image benign and malignant tumors, inflammatory diseases, and nail and scalp entities [171,172]. At its highest resolution, HFUS can image individual layers of the epidermis and dermis, cutaneous appendages, blood vessels and blood flow characteristics (with the color Doppler capability), and the stage of the disease (proliferation or regression) [172,173], and can be used for evaluating primary melanoma lesions, satellite/in-transit metastasis, and lymph node metastasis [174]. Melanoma typically presents as hypoechoic and or heterogeneous oval-shaped, fusiform, and hyper-vascularized structures with sharp margins and infiltration to the dermis [168]. The Doppler imaging technique shows increased and anarchic vascularization [168,174].

Importantly, HFUS does not rely on melanin as a contrast agent and is useful for detecting amelanotic melanomas [175]. HFUS with color Doppler imaging was used to investigate intralesional vascularization in 107 pigmented lesions and provided 100% specificity (albeit 34% sensitivity) in distinguishing pigmented melanomas from non-melanoma lesions [176]. Beyond its use for tumor screening, HFUS’s potential to provide deep penetration is a distinct advantage over other imaging modalities such as RCM (<0.3 mm) and OCT (~1–2 mm) and is important in estimating tumor (Breslow) thickness [169,172,177,178,179]. In one study, HFUS’s capability to determine tumor thickness showed a 99.4% correlation with histologic analysis for superficial spreading melanoma and a 98.4% correlation for nodular melanoma [180]. A study of 25 patients with cutaneous melanoma by Kunte et al. used B-mode HFUS for the preoperative identification and characterization of sentinel lymph nodes (SLN) to correctly identify two of the six positive SLNs, with a sensitivity of 33.3% and specificity of 100%, concluding that HFUS cannot replace SLN biopsy in the detection of micrometastis [181]. In a separate study evaluating melanoma surveillance and regional lymph node involvement, HFUS achieved a specificity of 85% to 99% and sensitivity of 95% to 100% [182]. Ultrasound coupled with the Berlin US Morphology Criteria and combined with fine needle aspiration cytology can significantly improve sensitivity [183].

The main advantage of HFUS is the capability to visualize the skin layers, deep structures, and perfusion patterns in real-time, allowing for the pre- and post-operative assessment of melanoma with a 3D image. However, HFUS cannot provide information about cellular morphological features due to its low resolution [184]. Additionally, HFUS is unable to differentiate between melanoma and inflammatory infiltrates as they are both hypoechoic, potentially resulting in an overestimation of tumor thickness when compared to histological section [169,185], although more recently, greater accuracy has been obtained [178,186]. Moreover, HFUS cannot detect pigment differences such as melanin content, as there are no specific pathogenic ultrasound features that are capable of distinguishing melanoma from nevi [170,187]. Additional limitations include measuring lesions that are less than 0.1 mm in depth, the detection of melanin, and the detection of flat epidermal lesions [187]. Finally, HFUS requires an operator extensively trained in its use and interpretation [172,188]. Images of US-guided fine needle aspiration and HFUS are included in Figure 3h,i.

Longport Inc., (Episcan I-200, Chadds Ford, PA, USA), FujiFilm VisualSonics (VevoMD, Bothell, WA, USA), and Cortex Technology (Aalborg, Denmark) are manufacturers of HFUS systems, among others.

### 4.8. Magnetic Resonance Imaging

Magnetic resonance imaging (MRI) has traditionally been utilized to image internal body structures, but in the last 20 years, it has been adapted in dermatology due to the development of specialized surface coils that enable higher image resolution than standard MRI coils [110,189]. With the use of contrast agents, MRI can differentiate the epidermis, dermis, and subcutaneous layers [190]. MRI has been used to image melanoma metastasis in vivo [191,192,193], as well as to image large skin cancers and their surrounding anatomy [194,195]. The significant advantage of MRI over most other imaging modalities is the exquisite soft-tissue contrast. The major disadvantages, however, are the low sensitivity, long scanning time, and requirement of exogenous contrast agents [196]. Furthermore, MRI cannot reliably differentiate between malignant and benign tumors. Nonetheless, a study by Jouvet et al. showed MRI to have a sensitivity of 84% and specificity of 87.1% for staging of melanoma [197]. Moreover, whole-body MRI is the imaging modality of choice for the detection of intracerebral and other distant metastases [198,199]. MRI images of a metastatic bone lesion in the left ilium are shown in Figure 3j, along with PET and CT images (discussed below). Manufacturers of MRI with dermatologic surveillance capabilities include GE Healthcare (Waukesha, WI, USA), Philips (Amsterdam, The Netherlands), and Siemens Healthcare (Munich, Germany), among others.

### 4.9. Raman Spectroscopy

Raman spectroscopy uses a laser to irradiate samples, which results in light scattering that varies depending on the molecular vibrations of the proteins and lipids making up the tissue [200,201]. A plot of the intensity of Raman scattered radiation as a function of its frequency difference from the incident radiation is called a Raman spectrum or the Raman shift [202]. Through inelastic scattering, the molecules within the tissue absorb photons from the light source and re-emit photons at a lower frequency [203]. Raman spectroscopy is a non-destructive and non-invasive method to image melanoma either in vivo or in excised tissue [98,200,204,205,206,207,208]. In a study by Lui et al., 518 skin lesions from 453 patients were imaged in vivo using Raman spectroscopy and the method was shown to distinguish malignant from healthy skin with a sensitivity of 95–99%, and a specificity of 15–54% [207]. Another study reported the correct classification of all melanomas with a specificity of 43.8%, sensitivity of 100%, and a number needed to treat of 2.7 [209]. For melanoma detection, Raman spectroscopy can differentiate melanomas, pigmented nevi, basal cell carcinomas, seborrheic keratoses, and healthy skin with a sensitivity and specificity of 85% and 99%, respectively; however, its imaging depth is limited, up to a few hundred micrometers, depending on the spectrometer setup [200,207]. Coherent anti-Stokes Raman scattering (CARS) microscopy has been utilized to detect pheomelanin signals in human tissue with amelanotic melanoma [210]. Another advance is the coupling of Raman spectroscopy with deep learning for melanoma detection, particularly for biopsied tissue analysis [200,204,205,206,209] (Figure 3k).

The Verisante Aura (Verisante Inc., Richmond, BC, Canada) is a commercially available handheld Raman spectroscopy device [98].

### 4.10. Elastic Scattering Spectroscopy

Elastic light single-scattering spectroscopy (ESS) is a technique that directs white light through a probe and measured the intensity at different spectra of the reflected light [211,212]. In this way, it operates similarly to Raman scattering spectroscopy. There is an FDA-cleared device by DermaSensor that includes an algorithm with an AI core, trained using over 11,000 spectral scans from 3500 skin lesions [213]. A multicenter trial was recently performed on the device involving 311 participants, including 44 melanomas. The device achieved 95.5% sensitivity and 32.5% specificity in a high-risk population with lesions selected for biopsy [214]. The technique has also been tested in a rodent model for its ability to monitor cancerous tissue response to laser therapy [215].

### 4.11. PET/CT and SPECT/CT

Positron emission tomography/computed tomography (PET/CT) is a whole-body imaging technique commonly used in the diagnosis of metastatic cancer. It relies on the introduction of a tracer, most commonly ^18^F-flouro-deoxy-glucose (^18^F-FDG), into the body. This tracer is a glucose analog with a positron-emitting radioisotope fluorine-18. Due to the increased glucose uptake of cancer cells, the tracer is visible more rapidly in malignant tissue than in healthy tissue [216]. Figure 3l shows a malignant lymph node detected by PET/CT. PET lacks optimal resolution to visualize early-stage cutaneous melanoma, and thus its use is limited to advanced metastatic melanoma and for staging clinically apparent nodal or distant metastasis (Figure 3j). PET has been tested for its ability for staging melanoma and to influence the treatment plan in melanoma patients with satellite or in-transit metastases, but showed only moderate sensitivity and specificity for this purpose [217]. Another application is for predicting or monitoring the therapeutic response in patients with metastatic melanoma [218,219]. ^18^F-FDG PET/CT parameters are promising predictors of the final response of metastatic melanoma patients to immunotherapy. In one study, 57 patients with metastatic melanoma were treated with ipilimumab or PD-1 inhibitors and received ^18^F-FDG PET/CT scans before treatment and 12–18 weeks later. The percent change in metabolic tumor volume and total lesion glycolysis were assessed [220]. The accuracy of parameters for therapy response were 96% in group 1 and 97% in group 2.

While PET/CT utilizes positron-emitting tracers and detects gamma rays produced by positron–electron interactions, single photon emission computed tomography/computed tomography (SPECT/CT) uses single photon-emitting tracers (detects gamma rays directly emitted by the tracers). While PET/CT is commonly used for diagnosing metastatic cancer, the most widespread use of SPECT/CT is to improve the accuracy of sentinel node biopsies for staging melanoma [221,222,223]. In a recent analysis of 1522 primary cutaneous melanoma patients, SPECT/CT was able to detect 50% more sentinel nodes than planar lymphoscintigraphy, which translated to a significantly reduced risk of death from melanoma. These findings have been replicated by others [224,225]. Figure 3m shows detection of SLNs by SPECT/CT.

Manufacturers of PET/CT and SPECT/CT systems include GE Healthcare (Waukesha, WI, USA), Philips (Amsterdam, The Netherlands), and Siemens Healthcare (Munich, Germany).

### 4.12. Lymphoscintigraphy

Lymphoscintigraphy for sentinel lymph node detection involves injecting a radiotracer, typically technetium-99m labeled colloids, into tissue near the identified melanoma. Then, the skin is massaged to accelerate the distribution of the tracer toward lymph nodes to identify a sentinel lymph node. The region is imaged using a gamma camera to detect the radiation emitted by the tracer. This process, which has been used in clinical practice for over 30 years, is often performed before sentinel lymph node biopsy [226,227]. More recently, a dye or another tracer is injected subsequently and combined with a handheld gamma camera for intraoperative monitoring during SLN biopsy. A typical image is shown in Figure 3n. There has been a concern regarding the possibility of false-negative sentinel node biopsies, and the quality of information from lymphoscintigraphy can vary from center to center [228]. Gamma cameras are available from many manufacturers, including Siemens Healthineers, GE Healthcare, and Philips Healthcare.

### 4.13. Non-Imaging (Genetic) Melanoma Detection and Disease Management

Genetic information from either skin cells or plasma are being employed to assist in melanoma detection and disease management. Epidermal genetic information retrieval (EGIR) is a melanoma diagnosis technology in which cells from the surface of pigmented melanocytic lesions are obtained via an adhesive patch and then sent to a lab for analysis [229,230]. A genomic signature is derived from the extracted mRNA, specifically analyzing the expression of two genes known to increase in melanoma: PRAME (preferentially expressed antigen in melanoma) and LINC (LINC00518, long intergenic noncoding RNA 518) [231]. EGIR is a diagnostic test intended to guide the decision on whether to biopsy or not and is especially useful for patients with multiple nevi or those that have a suspicious lesion on a cosmetically sensitive area [232,233]. Post biopsy, the gene expression profile (GEP) test is a prognostic test that utilizes reverse transcription polymerase chain reaction (RT-PCR) in primary tissue to determine the expression of thirty-one genes (three control) to predict metastatic risk [234,235]. For metastatic melanoma, liquid biopsies contain information on circulating melanoma cells (CMCs) and circulating tumor DNA (ctDNA). CMCs can be analyzed through the FDA-cleared Cell-Search platform, and ctDNA can be profiled using next generation sequencing (NGS) droplet digital PCR and customized genetic analysis [236]. Given the diversity of presentations of melanoma, these genetic assays can assist in disease management but have not yet delivered definitive information in most cases.

A 2017 validation study using the EGIR test examined 398 biopsied lesions, concluding the two-gene signature identified melanomas with a 91% sensitivity and 69% specificity [233]. Another study indicated that molecular expression profiling was able to differentiate melanoma from nevi with a sensitivity of 97.6% and specificity of 72.7%, suggesting this may be a useful method to reduce unnecessary biopsies [237]. In a cohort study of 205 stage I and II melanomas by Ferris et al., the post-biopsy GEP was able to predict distant metastasis with a sensitivity of 79% and specificity of 68% [235]. However, a 2023 panel from the Melanoma Prevention Working Group did not reach consensus on the role of gene expression profile testing for melanoma screening [238].

Genomic testing offers a method to possibly forego a skin biopsy, or SLNB, and can improve accuracy when diagnosing melanoma and determining the likelihood of metastasis [233,239]. Genomic testing may also aid in targeted therapy for stage III melanoma and higher, as cases displaying the BRAF-V600 mutation have proved to be somewhat treatable [240], especially through combination therapies [241]. However, the use of liquid biopsies for monitoring therapy response, cancer progression, and the onset of resistance is still experimental [236,242]. One of the companies that produces EGIR is Dermtech, Inc. (La Jolla, CA, USA). Two molecular tests for melanoma based on gene expression have been developed that acquire RNA from formalin-fixed paraffin-embedded sections rather than in situ lesions. GEP is offered by Castle Biosciences (Friendswood, TX, USA) and Myriad Genetics (Salt Lake City, UT, USA) offers myPath to distinguish nevi from melanoma [229]. CellSearch (Menarini Silicon Biosystems, Bologna, Italy) is the only FDA-approved system for detecting CMCs and several ctDNA based diagnostic assays have been FDA-approved, including Signatera (Natera, Austin, TX, USA).

### 4.14. The Role of Artificial Intelligence in Melanoma Technologies

AI has distinct and complementary applications for improving melanoma management which can be grouped as follows: image enhancement, image interpretation, and multimodal data integration. Image enhancement can be accomplished through AI-based super-resolution methods that use deep learning models to intelligently add details, textures, and sharper edges while minimizing noise [246,247]. These methods can improve resolution or reduce the time needed to acquire high-resolution images [248]. Image enhancement also has the potential to overcome difficulties in inter-operator variability [249]. AI can compensate for different conditions including lighting, steadiness of the hand of the operator, angle of probe used, and distance between imaging probe and skin. Enhancements could be accomplished by advances in signal and image processing, leading to standardization after relatively minimal training has been accomplished. However, such compensation models are in their infancy and would require a significant commitment from the research community. AI-based automatic image interpretation can boost melanoma detection, screening, and monitoring through methods such as pattern recognition, classification, object detection, segmentation, textural analysis, and feature extraction [250]. Pattern recognition and classification using methods such as convolutional neural networks [251] and transformers [252] are most effective for modalities with very large datasets, such as dermoscopy [253,254,255] and histology [256]. A shortcoming of the AI-based analysis of dermoscopy images that applies to rarer forms of melanoma and for melanoma in persons of color (where melanoma is far less prevalent) is that most studies have access to an insufficient number of training images for accurate model-building in these conditions [257]. Newer or emerging technologies generally implement methods that are leaner and can train from fewer samples, such as feature extraction [135] and textural analysis [258,259]. Finally, multimodal data integration techniques such as feature-level fusion, decision-level fusion, mixture of experts, and attention-based methods can be employed to incorporate dermoscopy, histopathology, ultrasound, or other imaging modalities together and/or to combine their results with genetic, molecular, and electronic health record data [260,261,262]. For example, one combination of modalities involves the use of RGB (dermoscopy) images (such as dermoscopy images) and extended near infrared images. A recent article demonstrated that training deep learning algorithms using both sets of images improved skin cancer diagnosis over RGB images alone [263]. Others have used AI to extract HSI information from RGB images, improving melanoma detection accuracy [264,265].

## 5. Technologies in Development for Non-Invasive Imaging of Cutaneous Melanoma

### 5.1. Photoacoustic Imaging (Optoacoustic Imaging)

**Figure 4:**
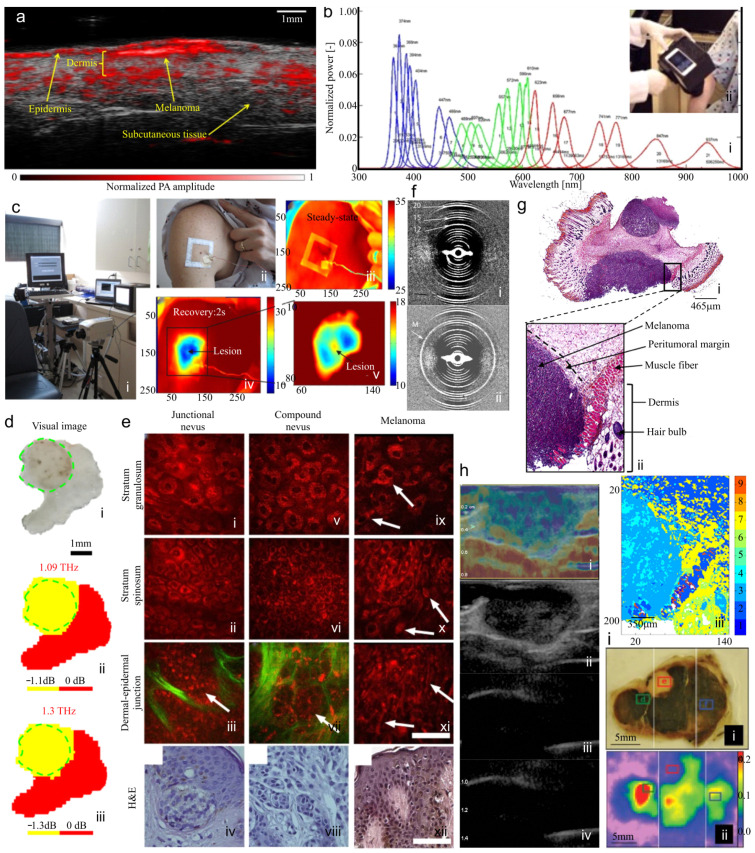
Images from technologies in development for cutaneous melanoma. (**a**) Photoacoustic imaging of in situ melanoma using linear array-based PAT, courtesy of [299]. (**b**) Hyperspectral imaging output for melanoma screening, courtesy of [304]. (**c**) Example of quantitative dynamic infrared imaging (**i**,**ii**). At steady state (**iii**), all images show similar heating, but at 2 s post recovery (**iv**), the melanoma has already cooled (**iv**,**v**), courtesy of [132]. (**d**) Terahertz pulse spectroscopy image of melanoma from mouse skin. Visual image (**i**), THz images (**ii**,**iii**) where red area corresponds to the normal region and yellow area corresponds to high THZ power-loss (indicative of melanoma), courtesy of [326]. (**e**) Multiphoton microscopy at different depths (rows) of junctional nevus (**i**–**iv**), compound nevus (**v**–**viii**), and melanoma (**ix**–**xii**). Cellular autofluorescence excited by MPM in red and collagen (from second harmonic generation signal) in green. Bottom row shows histology, courtesy of [333]. (**f**) Fiber diffraction of skin samples from (**i**) healthy control and (**ii**) patient with melanoma. M ring indicates additional ring not seen in healthy control, courtesy of [337]. (**g**) FTIR analysis of skin tissue compared with H&E staining (**i**) of insert, selected ROI for FTIR imaging (**ii**), FTIR reconstructed image using 9 classes of wavelengths reveal histological features (**iii**), courtesy of [345] (**h**) Real-time elastography shows stiff skin lesion (**ii**–**iv**), which on biopsy (**i**) was found to be melanoma, courtesy of [348]. (**i**) EPR analysis (**i**) Image of melanoma shown, cut in three, (**ii**) samples measured by EPR and images joined together. Boxes show highly pigmented, moderately pigmented, and nonpigmented areas (left to right), courtesy of [355].

In photoacoustic imaging (PAI), also known as optoacoustic imaging, the thermoelastic expansion of tissue chromophores occurs when they are irradiated by a nanosecond pulsed laser—resulting in the emission of acoustic waves that are detected by ultrasound transducers and translated into an image by a reconstruction algorithm [107]. To increase the specificity of PAI, disease-specific biomarkers have been utilized with endogenous absorbers, such as myoglobin, melanin, water, DNA, RNA, lipid, carboxyhemoglobin, bilirubin, cytochrome C, and methemoglobin [266]. Additionally, PAI has been used in conjunction with exogenous contrast agents such as nanoparticles and dyes [267,268,269,270,271,272]. With the use of a near-infrared light source, one can increase the penetration depth since the light absorption and scattering properties of hemoglobin and deep tissues are low at these wavelengths [271,273,274]. Because melanin is an endogenous contrast agent, PAI has great potential for melanoma assessment [34,268,275,276,277,278,279,280,281,282,283]. In fact, PAI has been used for detection and depth measurement [284], lymph node metastasis detection (both in vivo [285,286] and ex vivo [287,288]), tumor angiogenesis for monitoring the spread of metastasis and treatment efficacy [289,290], the detection of circulating tumor cells either in vivo [291,292] or by using photoacoustic flow cytometry [293,294], and virtual histology, using a technique called photoacoustic remote sensing [295,296]. PAI has been utilized to detect blood vessels within the skin as well as circulating tumor cells (CTCs) in the blood and for locating sentinel lymph nodes for biopsy. A PAT cross-sectional image of a melanoma is shown in Figure 4a.

PAI has been developed in both tomography (larger penetration depth with coarser resolution) and microscopy (shallow penetration depth, higher resolution) configurations [297,298]. While tomography is often employed for in vivo applications, recently, handheld photoacoustic microscopy devices have been developed that measure tumor thickness in patients with suspected melanoma lesions up to 7 mm [299,300]. In a study involving 27 patients with pigmented cutaneous lesions, the measured lesion thickness gave a high correlation to the resected surgical samples (*r* = 0.99, *p* < 0.001 for melanomas, *r* = 0.98, *p* < 0.001 for nevi), showing linear-array PAI is reliable in measuring the thickness of cutaneous lesions in vivo [299]. The exquisite ability of PAI to detect endogenous melanin with high accuracy is the reason PAI has been tested for so many melanoma-related applications.

PAI typically utilizes a transponder in contact with the medium to record and analyze acoustic waves. Non-interferometric PA remote sensing (PARS) microscopy analyzes the modulation of the elasto-optical refractive index caused by photoacoustic transients, when the absorbing interface has an appreciable refractive index difference from baseline [301]. This enables non-contact photoacoustic microscopy [301,302,303]. A non-interferometric approach with a low-coherence probe beam can be used to detect intensity variations unaffected by phase modulations. The system’s use has been demonstrated on a chicken model of melanoma [301]. PARS microscopy has also been applied on histology slides to enable the direct visualization of subcellular morphology on unprocessed, excised tissue, obviating the need for tissue freezing and histochemical staining [296]. A more in-depth discussion of PAI for melanoma detection and assessment can be found in Fakhoury et al. [34].

The main challenges of using melanin as the endogenous marker for melanoma (for detection and depth measurement, SLN analysis, and CTC assays) is that the use of such imaging modalities requires additional processing steps to differentiate melanoma from benign nevi (as both can have significant melanin), and they have no effective method to detect amelanotic melanoma. Additionally, PAI does not have adequate resolution to visualize cellular structures. One commercially available PA device is the Acuity Echo (iThera Medical, Munich, Germany); no commercially available devices have been developed for PARS.

### 5.2. Hyperspectral Imaging

Hyperspectral imaging (HSI) combines optical spectroscopy with optical imaging to analyze and record a larger optical spectrum for every pixel in the field of view (Figure 4b) [304]. Each pixel of the image contains spectral information, which is added to the two-dimensional spatial image, generating a three-dimensional data volume [305]. HSI identifies spectral characteristics, defined as the relationship between the wavelength and the physical properties of an object. This system typically uses a light source and a spectrometer, which records the scattered photons from a tissue’s surface up to 2 mm deep and presents an image. The advantage of HSI over MSI systems is that it can distinguish hemoglobin from melanin, providing more accurate information in pigmented lesions and darker skin types [306,307]. A recent study analyzed 325 lesions from 285 patients. Lesions were imaged prior to excision and a deep neural network algorithm was trained to distinguish between histopathologically verified malignant and benign lesions, to classify subgroups, and to delineate the extent of tumor [308]. Using the “majority vote” classification method, a sensitivity of 95% and specificity of 92% were achieved in differentiating malignant from benign lesions. HSI has also been used ex vivo to analyze pathological sections. Combining spectral features in the 500–675 nm band, a classification procedure achieved 98% accuracy [309]. HSI is most often used in combination with machine learning/deep learning techniques [310]. Another implementation of HSI does not require a spectrometer, but instead has been implemented by extracting color bands from calibrated RGB images via snapshot hyperspectral conversion [311] or by a CNN-based approach [265]. This technique shows significantly improved accuracy over the analysis of the RGB images alone at detecting melanoma and differentiating melanoma subtypes. Using the snapshot conversion method, one model, implemented with machine-learning analysis, detected nodular melanomas with a 0.9 sensitivity and 0.851 precision [311].

A commercially available SkinSpect™ dermatoscope (Spectral Molecular Imaging Inc., 201 N. Robertson Blvd, Beverly Hills, CA, USA) has been introduced that combines polarization and HSI to map the distribution of skin melanin and hemoglobin [307]. Another HSI system is the GaiaMicro-G-V10E-AZ4 (Dualix Spectral Imaging, Wuxi, Jiangsu, China), which is only approved for research. Any photographic system could be adapted for HSI using one of the methods described above, but based on their wide spectral acquisition set, smartphones have also been proposed as instruments for HSI-based melanoma detection [312].

### 5.3. Quantitative Dynamic Infrared Imaging (Thermographic Imaging)

Under certain conditions, the thermal radiation emitted by the skin in the infrared domain can be measured [62,132,313,314]. Due to increased blood supply, cancerous lesions, including melanoma, are warmer than healthy skin, and this difference in temperature can be detected by thermographic imaging [62,132,315]. Infrared imaging can image large surface areas and multiple lesions. Infrared thermography alone cannot distinguish between different types of cutaneous cancers; the purpose of this technology is to identify potential abnormalities. While passive thermography can provide only qualitative results, by adding a cooling system, the quantitative analysis of the efficiency with which an object radiates thermal energy (emissivity) can be determined (Figure 4c). Quantitative dynamic infrared imaging can possibly stage melanomas based on temperature differences and other thermal characteristics [62,316].

In a study of 74 patients, 251 palpable lesions were imaged with infrared thermography to determine if they were melanoma or non-melanoma, with a sensitivity ranging from 39 to 95% and specificity from 89 to 100%. Larger melanoma lesions (<15 mm) were more accurately detected via infrared thermography as hyperthermic [317]. Another study of 140 patients to detect skin cancer using quantitative dynamic infrared imaging demonstrated >99% sensitivity and >90% specificity (fifty-eight subjects were diagnosed with a cutaneous malignancy via biopsy, six of which had melanoma) [318]. A further refinement analyzed temperature recovery curves of suspicious lesions, achieving a sensitivity of 98% and specificity of 95% on a dataset of the same 116 subjects [319]. One of the manufacturers of infrared cameras used for thermography is QmagiQ, LLC (Nashua, NH, USA). Differences in skin temperature between cancerous and non-cancerous lesions can also be detected without the use of infrared cameras. One study showed that absolute skin surface temperatures and thermal conductivity (measured with a pen-shaped, guard-heated thermistor probe) differ between invasive melanoma vs. healthy skin or melanoma in situ [320].

### 5.4. Terahertz Pulsed Imaging

Terahertz pulsed imaging (TPI) is a non-invasive optical imaging modality using terahertz (THz) radiation [321,322]. The THz frequency range is 0.1 THz to 10 THz, corresponding to a wavelength range of 3 mm to 30 µm [323]. The frequency excites molecules, leading to vibrations which provide spectroscopic feedback and imaging contrast. THz radiation is highly absorbed by liquid water, which allows for imaging based on differences in water content between pathologic and non-pathologic skin lesions. The potential for imaging is limited by the water absorption coefficient of 80–350 cm^−1^ at 0.1–2.0 THz to a depth of 0.2–0.3 mm [322]. Sim et al. utilized TPI for the diagnosis of oral melanoma ex vivo in a frozen section. This research found significantly less water content in the melanoma versus healthy skin, allowing for differentiation between benign and malignant mucosal cells. Due to the increased protein and cell density in melanoma, the absorption coefficient and refractive index of the tumor are higher in diseased tissue than in healthy tissue [323]. TPI has been used for the in vivo imaging of dysplastic and non-dysplastic nevi, as it is effective at defining rough tumor margins (Figure 4d); however, penetration is better with frozen sections [322,324,325]. Promising terahertz imaging techniques include THz pulse imaging, continuous-wave terahertz, THz time-domain spectroscopy [326], and coherent THz confocal imaging [327]. The Terasense Group, Inc. (San Jose, CA, USA) produces Terahertz imaging devices.

### 5.5. Multiphoton Imaging

Multiphoton imaging is a spectrum of modalities that includes two-photon excitation microscopy (2PE), multiphoton microscopy (MPM), and second harmonic generation microscopy (SHG) [328,329]. Two-photon excitation signals are induced from endogenous fluorophores such as nicotinamide adenine dinucleotide (NADH), melanin, elastin, porphyrins, and collagen using two near-infrared lasers: the returning signal is captured by multichannel detectors and allows for subcellular resolution (0.5 µm lateral and 1–2 µm axial) [329,330,331] and results in minimal scattering. The multiphoton absorption also helps to suppress the background signal. This modality can be used to image tissue at a depth of up to 200 µm [330]. In SHG microscopy, the laser source generates a second harmonic signal when a laser passes through certain organic materials. The generated signal is twice the frequency (half the wavelength) of the incident laser and can be picked up by a detector. The signal varies depending on the characteristics of the tissue specimen. Many biologic components, including collagen, myosin, and microtubules, generate strong SHG signals [332]. MPM and SHG images of benign nevi and melanoma at different depths are shown in Figure 4e. A study conducted by Dimitrow et al. using multiphoton laser tomography in vivo to investigate eighty-three melanocytic skin lesions revealed distinct morphological differences in melanoma compared with melanocytic nevi. The study showed sensitivity values ranging from 71 to 95% and specificity values ranging from 69 to 97% [329]. Elagin et al. combined 2PE with optical coherence angiography to study equivocal melanocytic lesions [333]. They utilized a multiphoton tomograph, the MPTflex (JenLab, Berlin, Germany), and evaluated malignancy features from the images. They also acquired images by optical coherence angiography (OCA) to identify microvascular networks. Two-photon excitation imaging was able to discriminate benign lesions from melanoma in situ and invasive melanomas but could not differentiate between in situ and invasive melanomas. However, OCA detected significant differences in the vascular networks of melanoma in situ compared with invasive melanomas. Combining results from both modalities by a discriminant function analysis enabled perfect separation on their small dataset (49 lesions).

Chernyavskiy et al. used 2PE and SHG in addition to 1PE fluorescence and 1PE reflectance to image melanoma in mice. The resulting images revealed the collagen network, which is useful to identify invasive tumor cells. The authors concluded that these techniques are suitable to evaluate tissue modifications secondary to clinical interventions [332]. Multiphoton microscopy detects NADH without contrast to provide information about the mitochondrial organization within skin cells. The fluorescent patterns differ between normal skin and skin cancer: this has been used to differentiate skin cancer from healthy skin [334]. SHG is currently most widely used for non-invasive assessments of mouse melanoma growth [335]. A recent study showed the use of 2PE with an appropriate photosensitizer that targets melanomas for photodynamic therapy and in vivo melanoma ablation, which was tested in a mouse model [336].

Limitations of this imaging modality include laser damage for melanin rich lesions, slow speed of imaging, and small field of view. Manufacturers of multiphoton imaging devices with dermatologic capabilities include Olympus Corporation (Tokyo, Japan), Sutter Instrument (Novato, CA USA), Thorlabs Inc. (Newton, NJ, USA), and MPTFlex (Jena, Germany), among others.

### 5.6. Fiber Diffraction

Fiber diffraction for the detection of melanoma uses X-ray diffraction to acquire structural information from the fibrous content of the lesion (Figure 4f). Because biological fibers are composed of elongated molecules that are naturally aligned in parallel, identifying changes in fiber diffraction patterns of skin can indicate melanoma [337]. Interestingly, fiber diffraction can uncover physical changes in the biopsied tissue of fingernails and skin of cancer patients [337,338,339]. Each cancer type has a specific ring pattern, and the presence of a particular pattern indicates there is cancer somewhere on the body [338]. However, these ring changes only indicate the presence of carcinoma, and the actual location must then be determined. A study conducted by James and Kirby on 296 blinded skin samples (including 52 from melanoma patients) in subjects aged from 18 to 90 indicated that the fiber diffraction of skin could be a diagnostic test for melanoma even if other types of cancers are present [337]. Encouragingly, fiber diffraction’s potential for any-type cancer detection was reinforced in a small study of 30 samples, which showed the presence of prostate cancer with 100% sensitivity and 99.2% specificity [339]. With further development, fiber diffraction could be a low-cost, minimally invasive method to detect melanoma using small-angle X-ray beamlines at synchrotrons or a rotating anode X-ray generator [337,338]. However, this technique has not reported recent developments, suggesting translational or other difficulties.

### 5.7. Fourier Transform Infrared (FTIR) Spectroscopy and Microspectroscopy

Fourier transform infrared (FTIR) spectroscopy starts with a source with a wide spectrum of IR light, which passes through an interferometer before reaching the sample, and then measures how much of the beam is absorbed by the sample. The interferometer sequentially blocks and transmits different wavelengths to acquire multiple absorption spectra, and the data from the different acquisitions are combined and processed to determine the specific absorption across the IR spectrum, typically 4000–500 cm^−1^ or 780 nm^–1^ mm. This spectral range is able to extract information on intramolecular bonds in organic molecules, which enables the extraction of unique molecular fingerprints of biochemical information from fluids or tissue samples. FTIR is most often performed ex vivo using one of three sampling modes: transmission, transflection, and attenuated total reflection. The choice of sampling mode depends on the tissue or fluid being evaluated. FTIR can be used to construct images of tissue or cell architecture [340,341]. Because cancerous tissue has a different vibrational mode compared to healthy tissue, FTIR spectroscopy has been shown to rapidly detect melanoma in tissue samples [341]. Interestingly, FTIR can detect metabolic discharges from cancerous tissue into body fluids (blood, saliva, etc.), this has been used to provide guidance for clinical assessment and may aid in the rapid detection of melanoma [341,342]. FTIR can be combined with a microscope device to provide spatially resolved information on biochemical content (Figure 4g). This is often referred to as FTIR microspectroscopy [343]. Wald et al. used FTIR spectroscopy to image 34 melanoma biopsies from sentinel lymph nodes and was able to recognize melanoma cells with 87.1% sensitivity and 85.7% specificity [344]. Tosi et al. analyzed paraffin-embedded skin samples of benign nevi and melanoma and was able to differentiate them through principle component analysis [343]. FTIR microspectroscopy has a modest lateral resolution (~20 μm). Recently, FTIR is more often used to measure changes in melanoma tissues treated with anti-tumor molecules [345]. For example, the metastatic potential of different cell lines from the same genetic material could be differentiated by attenuated total reflection FTIR [346]. Manufacturers of FTIR spectroscopy systems include Thermo Fisher Scientific (Waltham, MA, USA), among others.

### 5.8. Real-Time Elastography

Real-time elastography (RTE) is an ultrasound imaging technique that provides images from the relative elasticity or stiffness of a lesion in comparison to adjacent healthy tissue [347,348]. Rigid tissue has less deformation than elastic tissue, and malignancy is indicated by a predominance of stiff tissue (Figure 4h) [348], which has been suggested for use for identifying tumor margins [348] and for differentiating between reactive and metastatic lymph nodes [349]. There are two diagnostic methods: elasticity score (ES) and strain ratio (SR). The limitation of RTE is that the technique is time-consuming and labor-intensive [350]. At a frequency of 14 MHz, RTE can penetrate up to 40 mm, which will allow the epidermis, dermis, and subcutaneous tissues to be imaged [351]. A metanalysis by Ying et al., which analyzed 835 superficial lymph nodes (LNs) for the diagnosis of malignant LNs, had an ES sensitivity of 74% and specificity of 90%, and an SR sensitivity of 88% and specificity of 81% [350]. In a study by Ogata et al., lymph nodes of 12 patients with cutaneous melanoma were imaged with RTE resulted in 100% sensitivity and 71% specificity when detecting metastasis with an ES cutoff score of three [349]. Hitachi, Ltd. (Tokyo, Japan) is one of the companies that produces a commercially available ultrasound platform with real-time elastography.

### 5.9. Electron Paramagnetic Resonance Imaging

Electron paramagnetic resonance (EPR) spectroscopy or electron spin resonance spectroscopy is a method for studying materials that have unpaired electrons (free radicals and paramagnetic transition metal ions). The basic concepts are analogous to NMR, but instead of measuring nuclear transitions in the nuclei, the spins excited are of the electrons. Melanin is one of very few organic compounds found in nature that contains unpaired electrons [352]; melanomas have strong EPR signals [353]. This has led to the suggestion that clinical low frequency EPR spectroscopy could be used as an in vivo technique for melanoma detection [354,355]. An EPR image of a melanoma is shown in Figure 4i. A first clinical trial of healthy volunteers and patients suspected of melanoma was performed, with subjects analyzed using a whole-body EPR scanner. The system was not sensitive enough to measure melanin differences in skin pigmentation, but was able to show the melanin signal was significantly higher in melanoma lesions than in benign nevi (*p* < 0.0001) [356]. However, this did not translate to high specificity. In addition, the system was not sensitive enough to detect Breslow depth with confidence. A shortcoming of this technique is it cannot see amelanotic melanomas. The system used was a whole-body clinical EPR system (Clin-EPR LLC, Lyme, NH, USA), operating at 1.15 GHz.

### 5.10. Multimodal Screening Technologies

The combination of two or more imaging systems into a single device can improve screening accuracy by leveraging the capabilities of both modalities. For example, there are reports of devices in which a Raman probe is incorporated onto a spectral domain OCT system [357,358], or a trimodal system including OCT, Raman, and PAI [359], or an ultrasound, OCT, and Raman combined system [360]. There is also a four-modal system combining OCT, Raman, ultrasound, and PAI [361]. In these cases, OCT and ultrasound are used for structural imaging (including tumor depth measurement), and PAI and Raman techniques are used for acquiring functional information [362,363,364,365]. We look forward to publications reporting observational trials of such multimodal systems to fully assess their capabilities compared with single-modal systems.

## 6. Remarks on Barriers to Technological Adoption

Barriers exist to introducing new screening technology to the dermatology clinic. So far, no melanoma screening technology has broken through and been embraced by the dermatology community. To accomplish this, new technology would need to achieve an appropriate sensitivity and specificity. Other practical considerations include whether the new system would take up valuable space in the clinic, perhaps needing a dedicated room, whether it would slow down clinical workflow, whether it is sufficiently durable (not requiring significant maintenance/calibration), and whether it would have burdensome training requirements for operation or interpretation of its results. Moreover, FDA approval of any new technology is essential before bringing a new device into clinical use, and insurance coverage is necessary to enable the clinic to recover the costs of purchasing and maintaining the equipment in a working condition (whether it needs daily or weekly calibration, for example, or whether it is costly to repair). Finally, some technologies are just not suited to melanoma detection and can never achieve sufficiently accurate specificity and sensitivity because they do not significantly assess the fundamental melanoma mechanism and therefore are not capable of analyzing the pathophysiology with sufficient clarity. These factors cause clinicians to be generally reluctant to replace or supplement dermoscopy and their own clinical expertise with new technology. In summary, key factors limiting the clinical use of these emerging modalities include cost (both capital cost and cost per use including specialist time for data interpretation), reimbursement, portability/lack of handheld devices, and lack of experience with interpretation of images.

Reviewing existing technologies with regard to these potential barriers to adoption, photography is a relatively inexpensive imaging method but can be cumbersome, lacks standardization with regards to color matching, which can be valuable for automated image analysis, has limited resolution without dermoscopy, and likely does not have the capability to analyze the pathophysiology sufficiently. Nevertheless, whole-body photographic imaging for screening, through companies such as MelanoScan, are marketed to patients at risk for skin cancer. The funding model is out of pocket payments for peace of mind. The use of photography coupled with smartphone apps has been described as lacking in consistent validation and transparent user communication [366]. Dermoscopy is the most inexpensive and prevalent technology in the clinic for assessing lesions, but its sensitivity, which is never very high, depends on the expertise of the user, and early and amelanotic melanomas are challenging to diagnose via dermoscopy, as they lack well-known superficial features of melanoma. In fact, there is a significant concern that dermoscopy will never have the capability to fully assess the pathophysiology of all forms of cutaneous melanoma.

The EIS system Nevisense is FDA-approved, reimbursement coverage is improving (covered by some private insurers and Medicare patients in about one-third of US states), and the device is fast, easy to use, and low-cost. One drawback is that while it enhances sensitivity, it may produce false positives, leading to unnecessary biopsies and patient anxiety. Another drawback is that it primarily assesses superficial lesions. SS-OCT provides around ten micron-resolution images to the depth of 2 mm, allowing for the assessment of tumors. However, although its use in dermatology is FDA-approved, it cannot reliably differentiate melanomas from benign pigmented lesions without the use of a sophisticated computational method, which has not yet been FDA-approved, so it is not currently marketed for melanoma detection. In addition, equipment costs are relatively high, and insurance does not reimburse for OCT skin imaging. Interestingly, insurance does not reimburse for OCT ocular imaging, but there, ophthalmologists appreciate the highly detailed images of the retina, optic nerve, and other eye structures, and offer the service to their patients (at increased cost). MSI was commercialized through the MelaFind device but failed commercially due to lack of clinical adoption. Moreover, its FDA approval was limited to “dermatologists experienced in melanoma detection”, narrowing its market potential, and it was never able to secure insurance reimbursement. HFUS has a good penetration depth and can visualize the size and shape of a tumor, but its poor resolution and low specificity make melanoma diagnosis difficult and there are no current efforts to commercialize it for melanoma screening. The Aura, which uses Raman spectroscopy, is an adjunct device that is approved for use in Canada but not in the US. The FDA previously required an endpoint sensitivity of 95% for a device that detects melanoma. Vita Imaging, which currently owns the patents to the Aura, expects FDA clearance in 2025. The ESS device DermaSensor is handheld and classifies lesions as “investigate further” or “monitor”. Notably, it was FDA-approved (in January 2024) for non-dermatologists, with the manufacturer targeting primary care physicians to use the device to improve their confidence and sensitivity for referring lesions to specialists. The current marketing model is to rent the devices for monthly fees (USD 399/month for unlimited users). RCM has the highest combination of resolution, sensitivity (88–98%), and specificity (57–98%), which can visualize cytological (nuclear and cytoplasmic) and architectural details of skin, similar to histology. However, it has failed to gain acceptance in the dermatology community. Non-technology-based barriers could include system cost, the low reimbursement rate, the cost to train users in acquiring images and interpreting them, and breaks in clinical workflow (patients typically must travel from dermatologists to separate centers for imaging). Moreover, neither RCM nor any of these other listed methods can stage melanoma (determine melanoma depth), so they are largely in the field of assistive screening technologies.

The technologies in development can be categorized as those solely applicable to melanoma screening and those with potentially wider applications. HSI, quantitative dynamic IR imaging, TPI, and EPR research are focused on developing melanoma detection systems. MPI is focused on biomedical research (mouse models of melanoma), FTIR shows promise for analyzing biopsied tissue, and RTE has been used to detect malignant lymph nodes, but the process is apparently time-consuming. Fiber diffraction research appears to be no longer active. PA microscopy and tomography has the widest range of potential applications for cutaneous melanoma [34], but questions remain whether PA applications can overcome the notably large potential barriers to clinical adoption.

Another barrier to clinical adoption that needs discussion is the difficulty of incorporating AI into medical devices in the US. AI has the potential to improve image resolution and standardize image interpretation, reducing training times and improving the specificity and sensitivity of nearly all imaging-based technologies. However, while there has been a significant surge in FDA approvals recently [367], currently, the FDA does not permit AI algorithms to be adapted after they have been released without further FDA review. More recently, the FDA has released guiding principles for developing predetermined change control plans for AI-enabled device software functions in order to support iterative improvement while maintaining device safety and effectiveness [368]. Their adoption would lead to improvements in a range of facets of melanoma care, from screening through post-treatment monitoring.

More different types of imaging and spectroscopic modalities have been researched for use with melanoma than nearly any other form of cancer, yet screening remains largely limited to dermatologists, often aided by dermoscopy. The ideal melanoma screening system should be highly accurate, low-cost, and sufficiently easy to use so that it can be available in every community, including in low-income and rural communities, since dermatologists can be hard to find in rural and low-income communities in the US and around the world.

## 7. Conclusions

Non-invasive imaging methods are continually being developed to improve melanoma screening, pathology, staging, detection of metastasis, and treatment monitoring. Novel methods demonstrate their value by showing improved accuracy at these tasks, which is an important starting point. On the other hand, the mere fact that very many different technologies and methodologies have been invented, developed, and promoted without being translated to the clinic highlights the fact that other considerations, beyond accuracy, are important for improving melanoma healthcare. Melanoma healthcare must start with effective screening to ensure that all melanomas are detected before they spread. To adequately detect melanoma, penetration depth should, at a minimum, reach the papillary dermis to differentiate melanoma in situ from invasive melanoma. Also, images with near-cellular-level resolution are desirable to distinguish histologic differences, enabling a definitive diagnosis of a lesion. Both RCM and OCT meet these two criteria. Increasing the opportunity to detect a malignancy at its earliest stage reduces the need for every other form of melanoma healthcare. Most existing and proposed screening imaging modalities are intended for dermatology clinics, which limit their availability. For example, they are not useful for screening people who cannot access dermatology clinics either because they cannot get an appointment or because of the lack of dermatologists in their community. Some screening modalities are targeted for use outside of dermatology clinics, but these must demonstrate high specificity to gain dermatologist (and patient) acceptance to minimize screening bottlenecks. In addition, many modalities, including Raman imaging, multispectral imaging, hyperspectral imaging, EIS, and ESS have very good sensitivity but are not able to provide visual evidence, which can reduce dermatologist acceptance. An ideal screening system would have to demonstrate high specificity and sensitivity, ease of use, and some form of imaging or other quantitative metric to increase dermatologist buy-in, and low capital costs to enable widespread implementation, but no current modality meets all of these criteria. After diagnosis, in order to characterize melanoma for staging, a depth penetration of at least 2 mm is required, recommending the use of HFUS or PAI. Then, for margin delineation for surgical planning, the penetration depth is important as well as a large field of view, making HFUS and PAI a good choice here as well. The detection of lymph node involvement has long been performed by lymphoscintigraphy, but SPECT/CT has recently demonstrated it can detect 50% more sentinel nodes than planar lymphoscintigraphy, reducing melanoma mortality. MRI and PET/CT are both used to find distant metastases, but PET/CT is the modality of choice to predict response to immunotherapies. Emerging technologies, while mainly focused on melanoma screening, also propose improvements for the staging and detection of distant metastases (PAI) and improved pathology (FTIR).

Table 1 summarizes all the modalities discussed in this review for detecting and managing melanoma, including how they are or could be used in different stages of melanoma healthcare, the type of biomarkers they provide, and the main limitations of each modality. A number of the modalities discussed in this review and included in Table 1 are focused on applications other than screening, seeking to replace current processes to reduce mortality and morbidity (detect metastasis sooner, find lymph nodes or circulating tumor cells with higher accuracy, better predict metastasis). These advances are also important, but likely will have to demonstrate not only improved accuracy but cost-effective clinical implementation to change current practices. In Table 1, each technology’s main utility is categorized as “prescreening”, that is, helpful prior to consultation with a dermatologist, “screening”, which happens in the presence of a dermatologist, “staging”, “surgical/treatment guidance”, “decision-making regarding treatment”, and “post-surgical/treatment monitoring”.

## Figures and Tables

**Table 1:** Imaging modalities and how they are or could be used in melanoma healthcare.

Imaging Modalities Currently Used in Medical Practice			
Technology	Biomarker	Main Applications	Main Limitation(s)
Smartphone/digital photography	ABCDE criteria.	Prescreening	Variable lighting, automated edge enhancement, user focusing decreases accuracy.
Total body photography	ABCDE criteria.	Prescreening	Detects changes over time, hence misses skin regions in genital, acral, scalp, within body folds. Large image files.
Dermoscopy	Irregular pigment network, asymmetrical structures, abrupt peripheral streaks, uneven color distribution within a lesion, and vascular features.	Prescreening/screening	Superficial assessment. Poor sensitivity for small melanomas, haphazard monitoring over time.
Electrical Impedance Spectroscopy	Melanomas show lower impedance at some frequencies due to higher water content and disrupted extracellular matrix and disrupted cell membranes. Melanomas have more pronounced change in impedance with change in frequency.	Prescreening/screening	Low specificity for melanoma, can be fooled by inflammation, ulceration, scar tissue. Requires trained physician to assess results.
Reflectance Confocal Microscopy (RCM)	Contrast is based on reflectance of different skin components. Melanin has a high refractive index and appears bright. RCM also detects irregularly shaped cells and disorganized arrangements of cells and structures.	Screening	Small field of view leads to lengthy imaging sessions for large lesions. Shallow imaging depth.
Optical coherence tomography (OCT)	Atypical cell structures and disorganized tissue architecture including honeycombed patterns, pagetoid spread, absence of dermal nests, and atypical melanocytes in the dermis. Also, optical features extracted and analyzed via machine learning. D-OCT: changes in microvascular structures in the skin including increased vascular density (angiogenesis), chaotic vessel architecture, irregular and dilated blood vessels, and irregular blood flow.	Screening	HD-OCT, OCM and LC-OCT have cellular resolution however do not have sufficient imaging depth for staging. Specificity and sensitivity have not been fully studied. D-OCT: focuses on blood flow changes caused by melanoma.
Multispectral Imaging	Differential absorption and reflectance of light at multiple wavelengths, capturing variations in melanin, hemoglobin, and oxygenation levels that distinguish malignant melanomas from benign lesions.	Prescreening/screening	Low specificity, cannot be used for rare melanomas.
Ultrasound (3.5–14 MHz)	Berlin morphology criteria for finding sentinel node metastases: peripheral perfusion, loss of central echoes and balloon shapes.	Staging, surgical/treatment guidance	May not provide reliable preoperative nodal staging.
High-Frequency Ultrasound (>15 MHz)	Hypoechoic lesions infiltrating the dermis from the epidermis. Anechoic content in suspicious lesions. Margins are usually sharp. Doppler shows increased and anarchic vascularization.	Staging	Allows deep penetration (1.5–8 mm) beneficial for estimating tumor thickness. Does not rely on melanin, thus useful for amelanotic melanoma. Poor sensitivity.
Raman Spectroscopy	Distinct spectral signature resulting from altered molecular compositions, such as elevated nucleic acids, proteins, lipids and melanin, reflecting the biochemical changes characteristic of malignant melanocytes compared to normal skin tissue.	Screening	Moderate specificity for in vivo screening.
Elastic scattering spectroscopy	Variation in light scattering patterns caused by differences in cellular and subcellular structures, such as nuclear size and density, which distinguish malignant melanoma from benign skin lesions.	Screening	Limited depth detection, high sensitivity but modest specificity.
Magnetic Resonance Imaging	Lesions are hyperintense by T1 due to reduced T1 relaxation time associated with melanin. T2 signal is reduced. T1 with contrast shows a heterogeneous peripheral rim enhancement.	Staging, surgical/treatment guidance	Low sensitivity, has long scanning times, and requires exogenous contrast agents.
Positron emission tomography/computed tomography (PET/CT)	18F-FDG (glucose analog that accumulates in cells with high metabolic activity).	Staging, surgical treatment/guidance, post-surgical/treatment monitoring	Ionizing radiation, requiring tracers, expensive.
Single Photon Emission Computed Tomography (SPECT/CT)	99mTc-labeled colloids/99mTc-Tilmanocept.	Staging, surgical/treatment guidance	Ionizing, requiring tracers, limited availability and cost, low spatial resolution.
Lymphoscintigraphy	99mTc-labeled colloids.	Surgical guidance	This nuclear medicine imaging technique, low spatial resolution, limited sensitivity for early-stage metastases, and lack of real-time imaging.
**Imaging Modalities Currently in Development**			
**Technology**	**Biomarkers**	**Proposed Main Utility**	**Strengths and shortcomings**
Photoacoustic Imaging (PAI)	Melanin is a strong endogenous chromophore, multispectral imaging enables visualization of melanoma-related angiogenesis, and other changes. Elevated optical absorption contrast of melanin at specific wavelengths, combined with increased vascularization and oxygenation heterogeneity that reflect the tumor’s metabolic and structural abnormalities.	Screening, staging, surgical/treatment guidance, post-surgical/treatment monitoring	Good depth penetration. Functional imaging (melanoma and hemoglobin). Versatile technique that can be implemented with either good depth penetration and/or high spatial resolution.
Hyperspectral Imaging	Unique spectral reflectance and absorption patterns across visible and near-infrared wavelengths, driven by variations in melanin concentration, hemoglobin content, and tissue morphology.	Screening	Portable imaging device. Can distinguish hemoglobin from melanin. Moderate specificity.
Quantitative Dynamic Infrared Imaging	Abnormal thermal signature and delayed heat dissipation patterns caused by increased metabolic activity and vascularization.	Screening	Can image large areas of skin rapidly. Low specificity, difficulty detecting small melanomas.
Terahertz Pulse Imaging	Altered terahertz refractive index and absorption coefficient, reflecting differences in water content, cellular density, and tissue composition between malignant melanomas and benign skin lesions.	Screening/possibly staging	Can define rough tumor margins but very limited depth detection. Poor sensitivity.
Multiphoton Imaging—Two Photon Excitation (2PE) Microscopy and Second Harmonic Generation (SHG) microscopy	Altered autofluorescence and reduced second-harmonic generation signals, changes in melanin distribution, collagen organization, and metabolic activity in malignant melanomas compared to normal or benign skin tissues. Reduced SHG signal intensity reflects structural abnormalities in the extracellular matrix associated with malignant melanoma progression.	Screening	Limited penetration (a few hundred µm) in depth penetration at subcellular (0.5 µm lateral and 1–2 µm axial) resolution.
Fiber Diffraction	Altered diffraction patterns of collagen and other fibrous proteins, indicating changes in molecular packing and structural organization associated with the tumor microenvironment in malignant melanoma.	Screening	Changes in fiber organization may occur in early-stage tumors. Poor sensitivity to cellular-level changes. Requires ordered molecular structures: fiber diffraction is best suited for analyzing highly ordered, periodic structures like collagen fibrils or keratin networks.
Fourier Transform Infrared Spectroscopy	Distinct absorption peaks corresponding to altered lipid, protein, and nucleic acid compositions, reflecting biochemical changes in malignant melanoma cells compared to normal or benign tissues.	Pathological staging	Rapid detection of melanoma in tissue samples through spectral changes. Not for in vivo use.
Real-time Elastography	Increased stiffness and altered elastic properties of malignant tissues, reflecting changes in extracellular matrix composition and tumor-induced mechanical heterogeneity.	Possibly screening, staging	Real-time imaging, excellent imaging depth up to 10 mm. Technique is time-consuming and labor-intensive. Poor specificity.
Electron paramagnetic resonance spectroscopy	The elevated levels of melanin-associated free radicals and altered paramagnetic properties, reflecting oxidative stress and metabolic changes characteristic of malignant melanoma.	Screening, staging	High quality 3-dimensional images, excellent penetration depth of 7 mm or more. Struggles with resolution and small melanomas.
